# Estimating global, regional and national rotavirus deaths in children aged <5 years: Current approaches, new analyses and proposed improvements

**DOI:** 10.1371/journal.pone.0183392

**Published:** 2017-09-11

**Authors:** Andrew Clark, Robert Black, Jacqueline Tate, Anna Roose, Karen Kotloff, Diana Lam, William Blackwelder, Umesh Parashar, Claudio Lanata, Gagandeep Kang, Christopher Troeger, James Platts-Mills, Ali Mokdad, Global Rotavirus Surveillance Network, Colin Sanderson, Laura Lamberti, Myron Levine, Mathuram Santosham, Duncan Steele

**Affiliations:** 1 London School of Hygiene and Tropical Medicine, London, United Kingdom; 2 Johns Hopkins Bloomberg School of Public Health, Baltimore, Maryland, United States of America; 3 Centers for Disease Control and Prevention, Atlanta, Georgia, United States of America; 4 University of Maryland School of Medicine, Baltimore, Maryland, United States of America; 5 Instituto de Investigacion Nutricional, Lima, Peru; 6 Vanderbilt University, Nashville, Tennessee, United States of America; 7 Christian Medical College, Vellore, India; 8 Institute for Health Metrics and Evaluation, Seattle, Washington, United States of America; 9 University of Virginia, Charlottesville, Virginia, United States of America; 10 World Health Organization, Geneva, Switzerland; 11 Bill & Melinda Gates Foundation, Seattle, Washington, United States of America; Universidad Nacional de la Plata, ARGENTINA

## Abstract

**Background:**

Rotavirus is a leading cause of diarrhoeal mortality in children but there is considerable disagreement about how many deaths occur each year.

**Methods and findings:**

We compared CHERG, GBD and WHO/CDC estimates of age under 5 years (U5) rotavirus deaths at the global, regional and national level using a standard year (2013) and standard list of 186 countries. The global estimates were 157,398 (CHERG), 122,322 (GBD) and 215,757 (WHO/CDC). The three groups used different methods: (i) to select data points for rotavirus-positive proportions; (ii) to extrapolate data points to individual countries; (iii) to account for rotavirus vaccine coverage; (iv) to convert rotavirus-positive proportions to rotavirus attributable fractions; and (v) to calculate uncertainty ranges. We conducted new analyses to inform future estimates. We found that acute watery diarrhoea was associated with 87% (95% CI 83–90%) of U5 diarrhoea hospitalisations based on data from 84 hospital sites in 9 countries, and 65% (95% CI 57–74%) of U5 diarrhoea deaths based on verbal autopsy reports from 9 country sites. We reanalysed data from the Global Enteric Multicenter Study (GEMS) and found 44% (55% in Asia, and 32% in Africa) rotavirus-positivity among U5 acute watery diarrhoea hospitalisations, and 28% rotavirus-positivity among U5 acute watery diarrhoea deaths. 97% (95% CI 95–98%) of the U5 diarrhoea hospitalisations that tested positive for rotavirus were entirely attributable to rotavirus. For all clinical syndromes combined the rotavirus attributable fraction was 34% (95% CI 31–36%). This increased by a factor of 1.08 (95% CI 1.02–1.14) when the GEMS results were reanalysed using a more sensitive molecular test.

**Conclusions:**

We developed consensus on seven proposals for improving the quality and transparency of future rotavirus mortality estimates.

## Introduction

Rotavirus is a leading cause of diarrhoeal mortality in children less than five years old (U5), but there is considerable disagreement about how many rotavirus deaths occur each year. Recent estimates from different sources range from ~120,000 to ~215,000 [1–3]. Accurate rotavirus mortality estimates help governments and donors prioritise public health interventions and provide a basis for assessing the impact of immunization on mortality rates. Conflicting estimates from different sources create confusion and can delay the introduction of important diarrhoea mortality prevention measures, such as rotavirus vaccines.

In recent years, three groups have produced estimates of rotavirus deaths:

1. CHERG—the Child Health Epidemiology Reference Group of the World Health Organization (WHO) and UNICEF. CHERG is now referred to as MCEE—the Maternal and Child Epidemiology Estimation group;
2. GBD—the Global Burden of Disease Study, a collaboration led by the Institute for Health Metrics and Evaluation (IHME); and,
3. WHO/CDC—the WHO and Centers for Disease Control and Prevention (joint estimates).

A meeting coordinated by WHO (Geneva, March 2015) facilitated the initial discussions on the differences between the currently available rotavirus mortality estimates. This work builds on a previous assessment of differences between CHERG and GBD estimates of all-cause U5 diarrhoea deaths [4]. Several gaps in the evidence were identified at an early stage in the process, and one important task was to conduct new analyses to help bridge these gaps. First, rotavirus is not associated clinically with acute bloody (dysenteric) diarrhoea and rarely with persistent diarrhoea (of 14 days duration or more). As a result, many of the rotavirus-positive proportions reported in hospital surveillance networks, and in the literature, exclude these cases, and simply report the rotavirus-positive proportion among hospitalised children with acute watery diarrhoea. If this proportion is applied to all episodes of diarrhoea resulting in hospitalisation, it will result in overestimates. Second, there is very limited evidence to inform whether the distribution of clinical syndromes for U5 diarrhoea hospitalisations (% acute watery, % acute bloody, % persistent) is similar to, and thus a reasonable proxy for, the distribution of clinical syndromes for U5 diarrhoea deaths. Most approaches assume that rotavirus-positivity among diarrhoea hospitalisations is a reasonable proxy for rotavirus-positivity among diarrhoea deaths. However, the two proportions are rarely reported in the same study population. Third, to date there has been no explicit quantification of the rotavirus attributable fraction among U5 diarrhoea hospitalisations, or the extent to which that varies depending on the type of diagnostic test used.

The aim of this manuscript is to compare the existing rotavirus mortality estimates, explain the reasons for differences, provide evidence to inform key areas of uncertainty, and propose improvements for future estimates.

## Methods

We used a range of methods and sources of data. First, we compared existing estimates of U5 rotavirus deaths at the global, regional and national level and identified key differences in the approaches used. Second, we used data from a large number of hospitals to estimate the proportion of U5 diarrhoea hospitalisations that were acute watery, acute bloody and persistent. Third, we used data from verbal autopsy studies to estimate the proportion of U5 diarrhoea deaths that were acute watery, acute bloody and persistent. Fourth, we calculated the proportion of U5 diarrhoea hospitalisations and U5 diarrhoea deaths that were rotavirus-positive in each of the African and Asian sites included in the Global Enteric Multicenter Study (GEMS). Fifth, we used data from GEMS to estimate the proportion of rotavirus-positive U5 diarrhoea hospitalisations that were entirely attributable to rotavirus, and quantified the increase in the rotavirus attributable fraction when a more sensitive molecular test was used to determine rotavirus-positivity.

All data used in this study were anonymized prior to access and analysis. Please see supporting information (S1 Table) for details about institutional ethical approvals, and how and where the data were collected.

### U5 rotavirus deaths: Comparison of estimates from GBD, CHERG and WHO/CDC

An independent reviewer (AC) compared the methods and data files published by the Global Burden of Disease 2013 Study (GBD 2013) [1, 5], CHERG [2, 6] and WHO/CDC [3, 7]. GBD provided a data file with country specific estimates of U5 rotavirus deaths [8].

We compared global, regional and national estimates of U5 deaths, U5 diarrhoea deaths and U5 rotavirus deaths for the year 2013 using a standard list of 186 countries (S1 File). CHERG did not report country estimates of U5 rotavirus deaths, so we multiplied country estimates of U5 diarrhoea deaths for the year 2013 by regional estimates of the proportion of U5 diarrhoea deaths due to rotavirus, as reported by CHERG for the year 2010. We removed two countries from the GBD list (Taiwan, Palestine) and seven from the WHO/CDC list (Cook Islands, Monaco, Nauru, Niue, Palau, St Kitts and Nevis, San Marino, Tuvalu) because they did not appear in both GBD and WHO/CDC datasets. GBD, CHERG and WHO/CDC used different classifications for grouping countries. For the purpose of this comparison exercise, all countries were grouped using the WHO classification system i.e. AFRO, AMRO, EMRO, EURO, SEARO, WPRO [9].

### Clinical syndromes of U5 diarrhoea hospitalisations: Acute watery, acute bloody, persistent

To estimate the proportion of U5 diarrhoea hospitalisations that were acute watery, acute bloody and persistent, we used data from 84 hospitals in 9 countries:

1. 50 hospitals (5 in Indonesia, 42 in Rwanda and 3 in Zambia) from the WHO-coordinated Global Sentinel Site Rotavirus Surveillance Network—GRSN [10];
2. 7 hospitals from the Indian National Hospital Rotavirus Surveillance Network—NRSN (Delhi, Hyderabad, Kolenchery, Ludhiana, Tirupati, Trichy and Vellore); and;
3. 27 hospitals included in the Global Enteric Multicenter Study—GEMS (1 in Bangladesh, 4 in India, 6 in Gambia, 10 in Kenya, 6 in Mozambique). Methods for recruiting and enrolling moderate-to-severe diarrhoea (MSD) cases in GEMS have been described in detail elsewhere [11]. We included 5 of the 7 GEMS sites in this particular analysis. Mali and Pakistan were excluded because they rarely hospitalised children [12].

To be included, sites had to be major paediatric hospitals or district hospitals with ≥100 children aged <5 years hospitalised for diarrhoea. For GEMS sites, inpatients included children with inpatient status at enrolment as well as children who were admitted after enrolment. Data had to be available for the full 12 months of the year (to account for rotavirus seasonality) and obtained before the introduction of rotavirus vaccination. Diagnoses were grouped into acute watery, acute bloody and persistent diarrhoea based on coding systems in place in the country and site. Acute syndromes were <14 days in duration, and persistent were ≥14 days. All GRSN, NRSN and GEMS sites excluded patients who were defined as “persistent” at the time of enrolment, but included patients who became persistent after enrolment. Paediatric logbooks were reviewed in the Indian NRSN sites and Indonesian GRSN sites. Cases were excluded (2% in India, 8% in Indonesia) if there was not enough information in the logbook to categorise them. Electronic discharge data were used in Rwanda and Zambia so all cases had to be coded into a category.

The Stata 14 command *metaprop* was used for meta-analysis of the proportion of U5 diarrhoea hospitalisations associated with acute watery diarrhoea (AWD), with random effects and exact confidence intervals [13].

### Clinical syndromes of U5 diarrhoea deaths

The clinical syndromes for U5 diarrhoea deaths were assessed using published verbal autopsy data from 5 demographic surveillance sites in Bangladesh, Ethiopia, Pakistan, Tanzania and Uganda [14] and verbal autopsy data from 4 sites in Cameroon, Malawi, Niger and Nigeria (Henry Kalter personal communication). The data came from investigation of child deaths identified in a household survey, including deaths in the community or a health facility, using the birth history method with follow-up questions to family members of the deceased child (S1 Table). All data sources included children aged <5 years and covered a period of at least 12 months, so they reflect all seasons.

The Stata 14 command *metaprop* was also used for meta-analysis of the proportion of U5 diarrhoea deaths associated with AWD, with random effects and exact confidence intervals [13].

### Rotavirus-positive proportion in U5 diarrhoea hospitalisations and U5 diarrhoea deaths in GEMS

We calculated the rotavirus-positive proportions among MSD cases aged <5 years who were admitted to hospital in 6 of the 7 country sites included in GEMS, using the conventional EIA (Enzyme immunoassay) test results. We excluded Mozambique because there was an unusually high number of positive samples in healthy controls, and only 55% of the rotavirus-positive cases and 7% of the rotavirus-positive healthy controls were shown to be rotavirus-positive on retesting with a different EIA test kit.

We calculated rotavirus-positive proportions separately for acute watery diarrhoea and all clinical syndromes combined (acute watery, acute bloody and persistent cases). In GEMS, if more than 9 children with MSD were identified in a fortnight, only the first 9 children were enrolled and tested for rotavirus; the remainder were recorded on a log and assumed to have the same rotavirus-positive proportion as enrolled cases that were identified in the same fortnight, age stratum (0-11m, 12-23m, 24-59m) and diarrhoea syndrome (acute watery, acute bloody, persistent).

We also calculated the proportion of U5 deaths that: a) tested positive for rotavirus within 7 days of death; and, b) had diarrhoea coded as the first or second cause of death on their verbal autopsy (VA) report. Rotavirus-positive children with a missing VA report (~20%) were assumed to have the same cause-of-death breakdown as rotavirus-positive children with a VA report.

For completeness, we also calculated the rotavirus-positive proportion among healthy controls as well as MSD cases that were not admitted to hospital.

### Proportion of rotavirus-positive U5 diarrhoea hospitalisations attributable to rotavirus in GEMS

GEMS tested for a wide range of enteric pathogens in the stools of MSD cases and healthy community controls without diarrhoea matched to cases by age, gender, and residence; controls were enrolled within 14 days of the index case. GEMS also included information about whether MSD cases were admitted to hospital or not.

We used multiple conditional logistic regression to calculate the odds ratio of rotavirus EIA positivity in hospitalized MSD cases vs matched healthy controls adjusted for the presence of other pathogens. All syndromes of diarrhoea were included. We then calculated the attributable fraction (AF) as described by Bruzzi *et al* [15]. These methods were the same as those used to estimate attributable fractions in the main GEMS analysis [12, 16]. However, we restricted the analysis to hospitalised cases, thought to be a better proxy for estimating rotavirus-attributable mortality than all MSD cases. We excluded Mozambique from all AF analyses due to concerns about the quality of the EIA testing, and did not estimate individual AFs for Mali and Pakistan because hospitalisation for diarrhoea was very rare in these sites.

Using these attributable fractions, which represent the fraction of hospitalised MSD cases with disease attributable to rotavirus, we calculated the attributable fraction among the exposed (AF_e_). The AF_e_ represents the fraction of rotavirus positive cases who have disease caused by rotavirus. The rotavirus-positive proportions used to derive the AF and AFe were based only on the children with MSD that were tested for rotavirus. These were age-specific (0-11m, 12-23m, 24-59m) and did not involve extrapolation to non-enrolled MSD cases.

Finally, we used previously described methods [17] to calculate the rotavirus attributable fraction based on quantitative Polymerase Chain Reaction (qPCR). We restricted the analysis to a subset of 721 hospital cases and matched controls, and calculated the AF for all country sites combined, excluding Mozambique. To quantify the test performance of EIA compared to qPCR, we repeated this analysis for EIA test results, and calculated the ratio between the two attributable fractions. All syndromes of diarrhoea were included. Confidence intervals were calculated by bootstrapping with 1000 iterations.

## Results

### Comparison exercise

GBD produce their own estimates of U5 deaths [18], whereas CHERG and WHO/CDC use U5 deaths from the UN Inter-agency Group for Child Mortality Estimation (IGME)[19]. Both GBD and IGME estimate approximately 6.3 million U5 deaths globally in 2013 (Table 1) but some important differences exist at country/regional levels e.g. ~739,000 (GBD) vs ~845,000 (IGME) in the Eastern Mediterranean Region (EMRO). The main methodological differences between GBD and IGME have been described in detail elsewhere and include the choice of data points selected (vital registration, census and household surveys) and fitting methods used [20].

**Table 1:** Comparison of CHERG, GBD and WHO/CDC estimates of U5 deaths, U5 diarrhoea deaths and U5 rotavirus deaths in the year 2013 by WHO region, and for selected large countries.

	GLOBAL	AFRO	AMRO	EMRO	EURO	SEARO	WPRO	Bangladesh	DR Congo	India	Indonesia
**U5 deaths**											
UN (IGME) used by CHERG	**6,282,254**	2,977,576	227,475	845,286	136,850	1,700,178	394,889	129,433	319,977	1,340,055	136,371
GBD	**6,271,643**	3,164,861	248,643	738,702	130,573	1,604,028	384,836	128,228	340,416	1,249,673	148,807
WHO/CDC	**-**	-	-	-	-	-	-	-	-	-	-
**Proportion of U5 deaths due to diarrhoea**											
CHERG	**0.09**	0.10	0.04	0.10	0.04	0.10	0.06	0.06	0.11	0.10	0.06
GBD	**0.08**	0.10	0.05	0.12	0.03	0.06	0.02	0.01	0.17	0.06	0.06
WHO/CDC	**-**	-	-	-	-	-	-	-	-	-	-
**U5 diarrhoea deaths**											
CHERG	**577,508**	293,289	9,297	84,592	5,689	162,298	22,344	8,298	33,730	140,451	7,505
GBD	**519,485**	312,297	11,923	88,071	3,694	94,574	8,926	1,715	57,344	80,188	8,694
WHO/CDC	**577,508**	293,289	9,297	84,592	5,689	162,298	22,344	8,298	33,730	140,451	7,505
**Proportion of U5 diarrhoea deaths due to Rotavirus**											
CHERG	**0.27**	0.27	0.23	0.31	0.26	0.26	0.33	0.26	0.27	0.26	0.26
GBD	**0.24**	0.24	0.18	0.18	0.26	0.27	0.42	0.12	0.13	0.26	0.37
WHO/CDC	**0.37**	0.39	0.26	0.36	0.31	0.35	0.43	0.33	0.40	0.34	0.50
**U5 rotavirus diarrhoea deaths**											
CHERG	**157,398**	78,601	2,176	26,477	1,473	41,386	7,284	2,116	9,040	35,815	1,914
GBD	**122,322**	73,758	2,178	15,984	976	25,637	3,790	202	7,523	21,205	3,176
WHO/CDC	**215,757**	115,023	2,455	30,577	1,752	56,287	9,664	2,723	13,526	47,082	3,771

Region and global estimates may differ from official WHO/CDC, CHERG and GBD estimates because a standard set of countries and regions was used and no rounding was done prior to aggregation.

**Fig 1:**
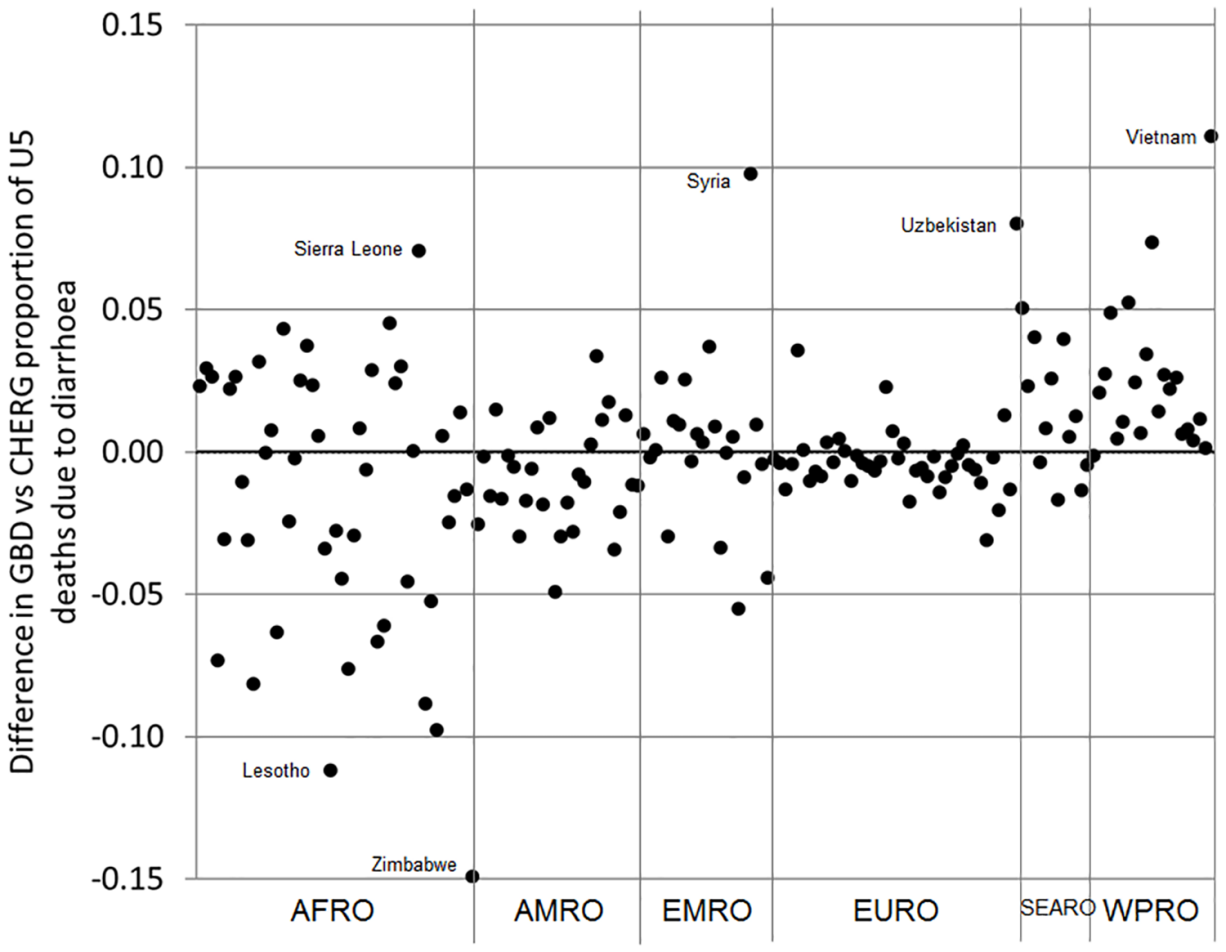
Country-level differences in GBD vs CHERG estimates of the proportion of U5 deaths due to diarrhoea in the year 2013 by WHO region.

GBD and CHERG produce their own estimates of the proportion of U5 deaths due to diarrhoea [5, 6]; WHO/CDC use the CHERG estimates. GBD and CHERG estimated that 8–9% of U5 deaths were caused by diarrhoea at the global level in the year 2013 (Table 1). Differences in GBD and CHERG estimates for the South East Asia (SEARO) region (6% vs 10%) are driven by differences in estimates for India (6% vs 10%) where U5 diarrhoea deaths are ~80,000 vs ~140,000 respectively (Table 1). In other regions there is more agreement. Estimates for the African (AFRO) region are consistent overall (10% vs 10%) but there are still large differences at country level e.g. Zimbabwe (Fig 1).

Methodological differences between GBD and CHERG have been described in detail elsewhere [4]. In brief, CHERG excluded verbal autopsy studies that only investigated a single cause of death and data points from incomplete vital registration systems in higher mortality settings. GBD included these data points and adjusted for missing data. GBD also included unpublished data points obtained under third party data use agreements whereas CHERG only use publicly available data points [21].

**Fig 2:**
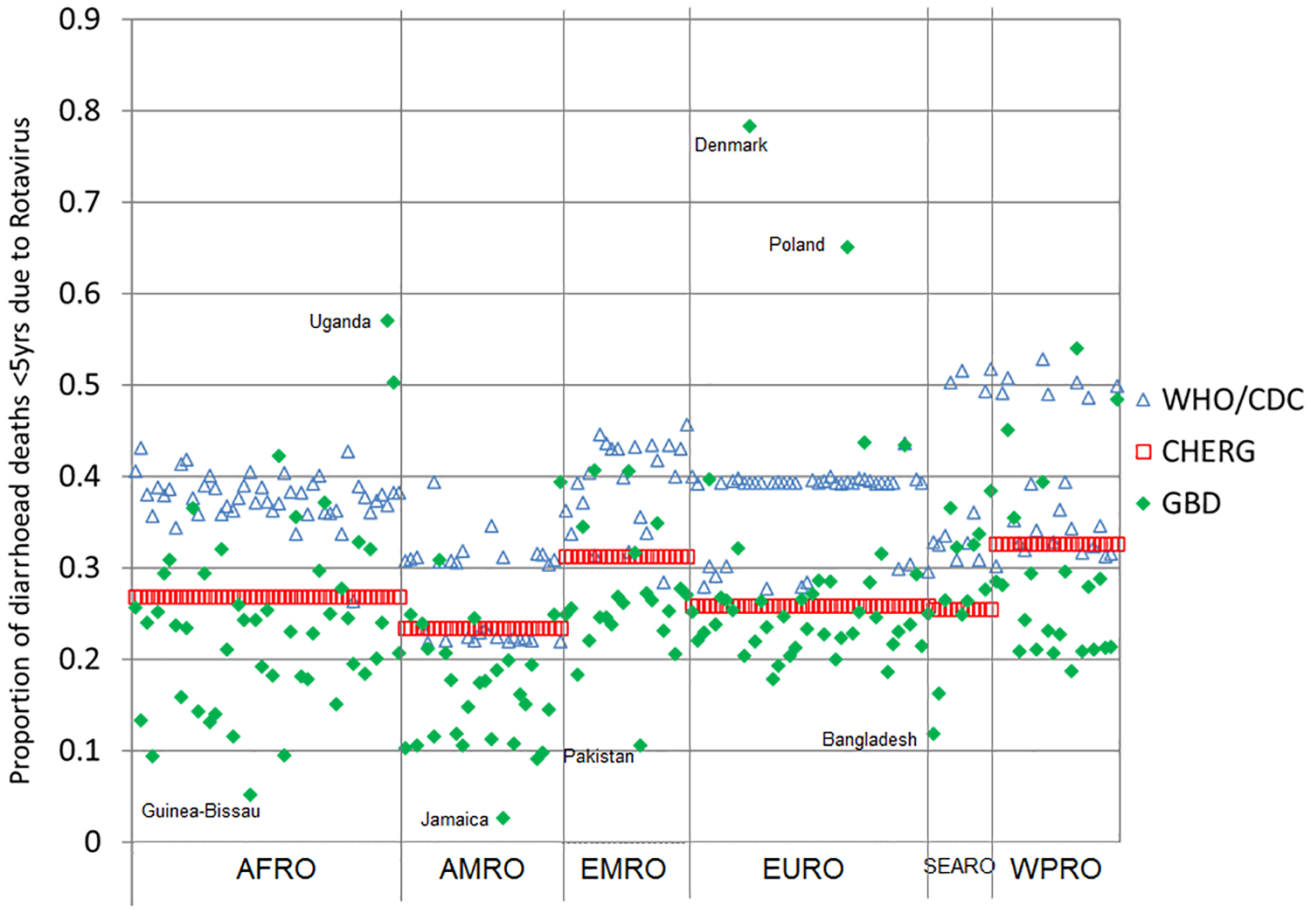
Country-level variation in the fraction of U5 diarrhoea deaths due to rotavirus in the year 2013 by source of estimates and by WHO region.

All three groups produce their own estimates of the proportion of U5 diarrhoea deaths that are attributable to rotavirus. For the year 2013, the global proportions were 24% (GBD), 27% (CHERG) and 37% (WHO/CDC). These correspond to 122,322 (GBD), 157,398 (CHERG) and 215,757 (WHO/CDC) U5 rotavirus deaths (Table 1). Fig 2 shows the extent of variation in the fraction of diarrhoea deaths attributed to rotavirus across countries within each WHO region. There are large differences in some countries; for example, in DR Congo the proportions are 13% (GBD), 27% (CHERG) and 40% (WHO/CDC).

The three groups used different methods to:

1. select data points (rotavirus-positive proportions);
2. extrapolate data points to individual countries;
3. account for rotavirus vaccine coverage;
4. convert rotavirus-positive proportions to rotavirus attributable fractions; and,
5. calculate uncertainty ranges.

A more detailed description of these differences can be found in the supporting information (S1 Appendix).

### Clinical syndromes for U5 diarrhoea hospitalisation

**Fig 3:**
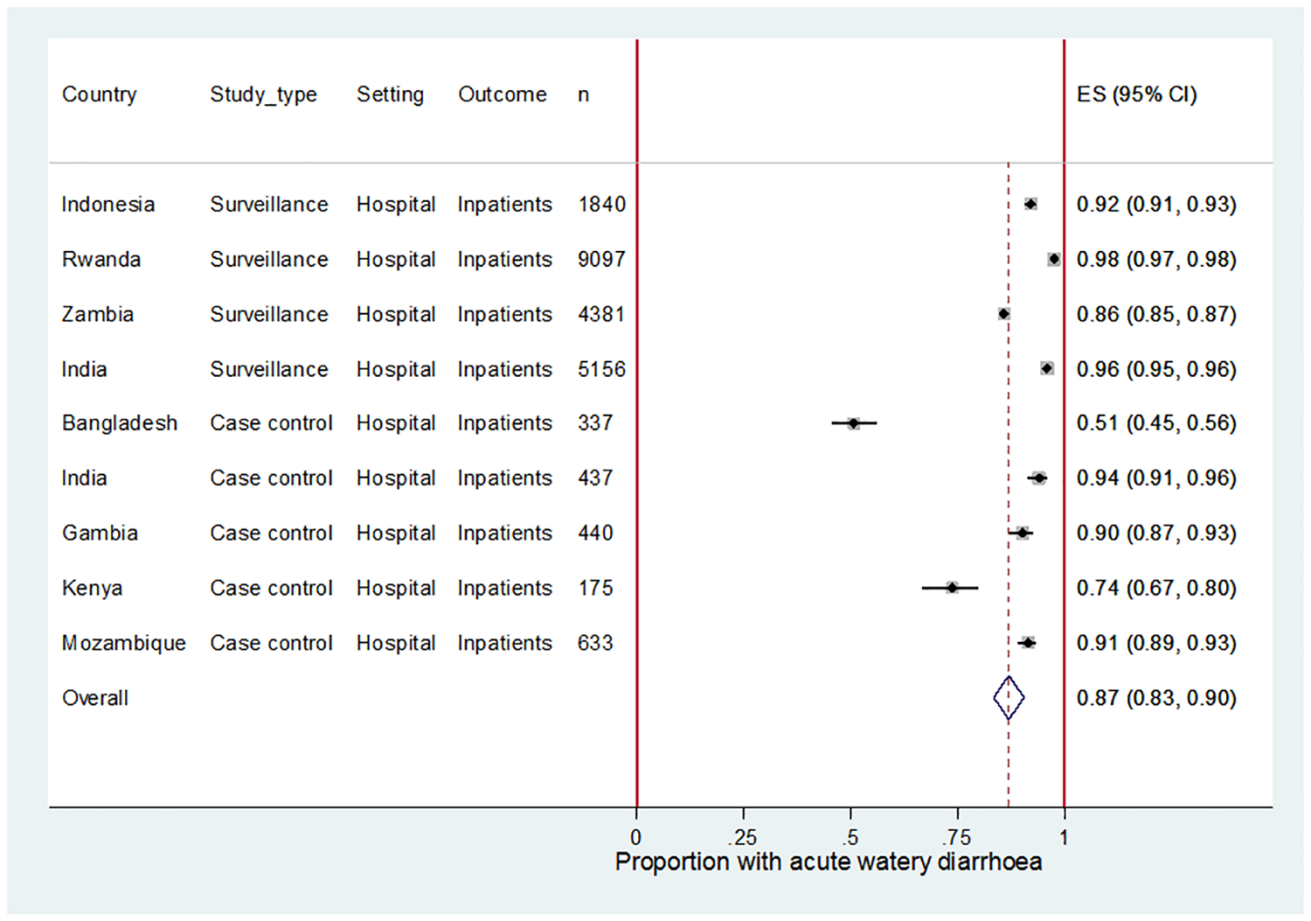
Meta-analysis showing the proportion of U5 diarrhoea hospitalisations associated with acute watery diarrhoea (AWD) for selected sites in Africa and Asia.

Table 2 shows the distribution of clinical syndromes for U5 diarrhoea hospitalisations for various sites in Africa and Asia. A meta-analysis including data from all GRSN, NRSN and GEMS sites suggests that acute watery diarrhoea was associated with 87% (95% CI 83–90%) of U5 diarrhoea hospitalisations (Fig 3) but there was substantial evidence for heterogeneity (I-squared 99.08%, p = 0.00) between the studies. The GEMS site in Bangladesh (Mirzapur) had a very high rate of acute bloody diarrhoea for reasons that are not clear.

**Table 2:** Number and proportion of acute watery, acute bloody and persistent cases among U5 diarrhoea hospitalisations and U5 diarrhoea deaths in various settings before rotavirus vaccine introduction.

Source	Study location	Study type	Study period	Diarrhoea outcome	Age	Total^Δ^ n	Acute Watery n	Acute Bloody n	Persis-tent* n	Acute Watery %	Acute Bloody %	Persis-tent %
**Clinical syndromes of U5 diarrhoea hospitalisations**												
GRSN	Indonesia	Surveillance hospitals (n = 5)	2014–15	Inpatients	<5yrs	1840	1695	110	35	**92%**	6%	2%
	Rwanda	Surveillance hospitals (n = 42)	2012	Inpatients	<5yrs	9097	8878	199	20	**98%**	2%	0.2%
	Zambia	Surveillance hospitals (n = 3)	2009–11	Inpatients	<5yrs	4381**	3761	15	-	**86%**	0.3%	-
NRSN	India	Surveillance hospitals (n = 7)	2013–14	Inpatients	<5yrs	5156	4940	196	20	**96%**	4%	0.4%
GEMS	Bangladesh	Case control study hospitals (n = 1)	2007–10	Inpatients	<5yrs	337	171	154	11	**51%**	46%	3%
	India	Case control study hospitals (n = 4)	2007–10	Inpatients	<5yrs	437	411	17	10	**94%**	4%	2%
	Gambia	Case control study hospitals (n = 6)	2007–10	Inpatients	<5yrs	440	397	14	28	**90%**	3%	6%
	Kenya	Case control study hospitals (n = 10)	2007–10	Inpatients	<5yrs	175	129	3	44	**74%**	1%	25%
	Mozambique	Case control study hospitals (n = 6)	2007–10	Inpatients	<5yrs	633	579	6	48	**91%**	1%	8%
**Clinical syndromes of U5 diarrhoea deaths**												
Verbal	Bangladesh	Demographic surveillance (16)	2003–11	Deaths	1-59m	59	43	7	9	**73%**	12%	15%
autopsy studies	Pakistan	Demographic & Health Survey (16)	2006–7	Deaths	1-59m	318	213	22	83	**67%**	7%	26%
	Cameroon	Subnational household survey***	2006–10	Deaths	1-59m	166	125	20	22	**75%**	12%	13%
	Ethiopia	Demographic surveillance (16)	2003–12	Deaths	1-59m	60	19	6	35	**32%**	10%	58%
	Malawi	Subnational household survey***	2008–11	Deaths	1-59m	149	118	18	13	**79%**	12%	9%
	Niger	Demographic & Health Survey***	2006–10	Deaths	1-59m	160	104	32	24	**65%**	20%	15%
	Nigeria	Demographic & Health Survey***	2009–13	Deaths	1-59m	537	435	70	32	**81%**	13%	6%
	Tanzania	Demographic surveillance (16)	2000–11	Deaths	1-59m	80	48	13	19	**60%**	16%	24%
	Uganda	Demographic surveillance (16)	2007–10	Deaths	1-59m	77	37	9	31	**48%**	12%	40%

^**Δ**^Totals for GEMS sites do not sum exactly due to rounding

*GRSN, NRSN and GEMS persistent cases include only those children who progressed to ‘persistent’ status (14+ days duration) after acute admission

** 605 cases in Zambia were classified as ‘non-infectious diarrhoea’, which is likely to include persistent cases as well as other cases that could not be classified as acute watery or acute bloody diarrhoea.

*** Henry Kalter, personal communication

### Clinical syndromes for U5 diarrhoea deaths

**Fig 4:**
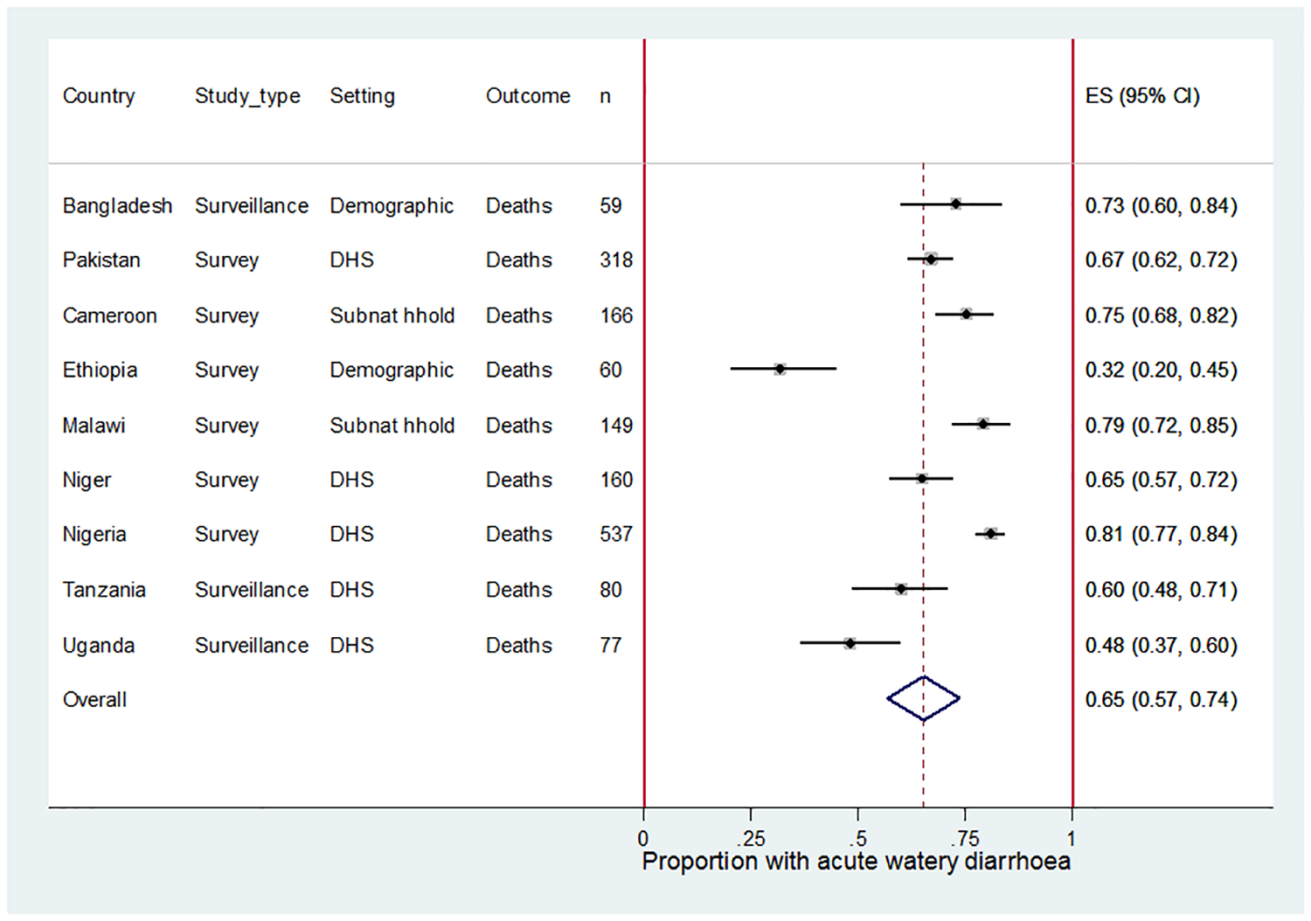
Meta-analysis showing the proportion of U5 diarrhoea deaths associated with acute watery diarrhoea (AWD) for selected sites in Africa and Asia.

Table 2 shows the distribution of clinical syndromes for U5 diarrhoea deaths. A meta-analysis suggests that acute watery diarrhoea was associated with 65% (95% CI 57–74%) of U5 diarrhoea deaths (Fig 4) but again there was substantial evidence for heterogeneity between the studies (I-squared 92.06%, p = 0.00). In four of the nine countries with verbal autopsy data, the clinical syndromes of diarrhoea deaths were compared for those who died in any type of health facility and those who died in the home, as reported by the family respondent. Most of deaths were in the home (Cameroon 70%, Malawi 50%, Niger 86% and Nigeria 78%) but the distribution of acute watery, acute bloody and persistent diarrhoea was similar irrespective of the place of death (Kalter, personal communication).

### Rotavirus-positive proportion in U5 diarrhoea hospitalisations and U5 diarrhoea deaths in GEMS

For all GEMS sites combined (excluding Mozambique), rotavirus was detected (EIA-positive) in 44% of acute watery U5 diarrhoea hospitalisations (55% in Asia, and 32% in Africa) (Table 3). When all clinical syndromes of diarrhoea were included, the rotavirus-positive proportion was 38% (44% in Asia; 30% in Africa).

**Table 3:** Number and proportion of rotavirus infections in healthy controls and different types of diarrhoea cases aged <5yrs in GEMS.

			Bangladesh	India	Pakistan	ASIA	Gambia	Kenya	Mali	AFRICA	TOTAL
Controls		Number positive	74	42	66	**182**	42	39	43	**124**	**306**
		Number tested	2465	2014	1838	**6317**	1569	1883	2064	**5516**	**11833**
		% Positive	3%	2%	4%	**3%**	3%	2%	2%	**2%**	**3%**
All syndromes	MSD	Number positive	23	292	187	**502**	208	250	573	**1031**	**1533**
	Not hospitalised	Number tested	741	1398	902	**3041**	1038	1644	3109	**5791**	**8832**
		% Positive	3%	21%	21%	**17%**	20%	15%	18%	**18%**	**17%**
	MSD	Number positive	111	233	2	**346**	152	40	5	**197**	**543**
	Hospitalised	Number tested	337	437	8	**782**	440	175	41	**656**	**1438**
		% Positive	33%	53%	25%	**44%**	35%	23%	12%	**30%**	**38%**
Acute watery	MSD	Number positive	0	284	125	**409**	198	207	515	**920**	**1329**
	Not hospitalised	Number tested	2	1315	485	**1802**	899	1258	2657	**4814**	**6616**
		% Positive	0%	22%	26%	**23%**	22%	16%	19%	**19%**	**20%**
	MSD	Number positive	93	230	0	**323**	141	33	5	**179**	**502**
	Hospitalised	Number tested	171	411	3	**585**	397	129	35	**561**	**1146**
		% Positive	54%	56%	0%	**55%**	36%	26%	14%	**32%**	**44%**
	Deaths	Number positive	4	0	0	**4**	1	2	5	**8**	**12**
	(within 7 days of enrolment)	*Number VA adjudicated*	4	0	0	**4**	0	1	2	**3**	**7**
		*Number VA confirmed as diarrhoea*	4	0	0	**4**	0	0	2	**2**	**6**
		*% VA confirmed as diarrhoea*	100%	-	-	**100%**	-	0%	100%	**67%**	**86%**
		Number positive (adjusted for VA)	4	-	-	**4**	-	0	5	**5**	**10**
		Total deaths	5	0	1	**6**	12	12	7	**31**	**37**
		% Positive	*	*	*	*	*	*	*	*	**28%**

*proportion positive not reported at country-level due to small numbers.

VA = verbal autopsy.

Rotavirus was detected (EIA-positive) in 32% (12/37) of children aged <5yrs that died within 7 days of enrolment at any type of health facility with acute watery MSD. Seven of the rotavirus-positive children that died had a VA report, and 6 of these children (86%) had diarrhoea coded as a primary or secondary cause of death. Assuming that children with rotavirus who lacked a VA report died from diarrhoea at the same rate as those with a report, the fraction of U5 diarrhoea deaths that were rotavirus-positive was estimated to be 28% (10/37) (Table 3). It was not possible to consider all syndromes because there were very few acute bloody diarrhoea deaths (n = 3) and no persistent cases were eligible for inclusion in this analysis due to the short 7-day follow-up period.

The rotavirus-positive proportion was 3% in healthy controls. The rotavirus-positive prevalence in MSD inpatients was approximately double the rotavirus-positive prevalence in MSD outpatients. 10% (147/1438) of inpatients and 20% (1782/8832) of outpatients had no detected pathogen using conventional testing methods.

### Proportion of rotavirus-positive U5 diarrhoea hospitalisations attributable to rotavirus in GEMS

The AFe value (equivalent to the rotavirus attributable fraction among rotavirus-positive U5 diarrhoea hospitalisations) was 0.97 (95% CI 0.95–0.98) for all included GEMS sites (Table 4) and all diarrhoea syndromes combined.

**Table 4:** Rotavirus positive proportion, attributable fraction (AF) and attributable fraction in the exposed (AFe) for MSD cases <5yrs that were hospitalised with all syndromes of diarrhoea in GEMS*.

	Rotavirus positive proportion *(includes extrapolation to MSD cases that were not enrolled)* Value	Rotavirus positive proportion (enrolled MSD cases only)** Value	AF		AFe	
			Value	95% CI	Value	95% CI
Bangladesh	0.33	0.35	0.34	(0.30, 0.38)	0.96	(0.94, 0.98)
India	0.53	0.48	0.47	(0.42, 0.52)	0.99	(0.97, 1.00)
Pakistan	0.25	0.22	***	***	***	***
**ASIA**	**0.44**	**0.40**	**0.39**	**(0.36, 0.42)**	**0.97**	**(0.95, 0.98)**
Gambia	0.35	0.29	0.28	(0.23, 0.33)	0.98	(0.96, 1.00)
Kenya	0.23	0.21	0.19	(0.13, 0.26)	0.91	(0.82, 0.99)
Mali	0.12	0.18	***	***	***	***
**AFRICA**	**0.30**	**0.26**	**0.24**	**(0.20, 0.29)**	**0.95**	**(0.91, 0.99)**
**ALL SITES**	**0.38**	**0.35**	**0.34**	**(0.31, 0.36)**	**0.97**	**(0.95, 0.98)**

* Includes all syndromes of diarrhoea i.e. acute watery diarrhoea, acute bloody diarrhoea, and acute watery cases that became persistent after enrolment.

** These values were based on the children that were enrolled i.e. tested for rotavirus. Age-specific presentations of these values (0-11m, 12-23m, 24-59m) were used in the calculation of AF and AFe.

*** Rotavirus-positive hospitalisation was rare in Mali (n = 5) and Pakistan (n = 2) so country-specific AF and AFe are not reported. The data for these countries are included in the estimates for ASIA, AFRICA and ALL SITES.

Using qPCR instead of EIA for rotavirus detection increased the AF by a factor of 1.08 (95% CI 1.02–1.14).

### Proposed improvements

We propose a number of improvements for consideration by all groups involved in the development of future rotavirus mortality estimates.

### Reporting a standard set of minimum variables to describe all input data points

Previous comparison exercises have stressed the need for input data points to be made available at the time estimates are published [4, 20]. Recent Guidelines for Accurate and Transparent Health Estimates Reporting (GATHER) have recommended publication of a spreadsheet table with details about the data points used to inform estimates [22]. These guidelines do not provide explicit guidance on the variables that should be reported. We suggest that the following standard set of minimum variables should be reported: (a) author/reference; (b) country; (c) sub-national location; (d) data collection period; (e) age range; (f) type of study; (g) type of diagnostic test; (h) number of enteric pathogens tested; (i) inpatient/outpatient; (j) pre/post implementation of rotavirus vaccine in the public sector, or preferably a more precise estimate of rotavirus vaccine coverage with details about the source of sub-national or national coverage data used; (k) type of clinical syndrome e.g. acute watery, all syndromes; (l) included/excluded in final estimates; (m) justification if excluded; (n) rotavirus-positive proportion (unadjusted); (o) rotavirus-positive proportion (adjusted); and, (p) description of adjustment applied. Inclusion and exclusion criteria should be clearly documented, and any exclusions applied after data extraction should be justified using a clearly defined framework for evaluating data quality and outliers.

### Annual online publication of WHO surveillance data points in spreadsheet format

A spreadsheet table should be published annually on the WHO web site to allow for potential inclusion of GRSN data by all groups in future estimates. At a minimum, rotavirus-positive proportions <5 years should be made available by country (aggregated across sub-national sites) and by calendar year. The standard set of recommended variables described above should be reported for each data point. To ensure the integrity and confidentiality of country surveillance data and to protect the ownership of those who collect the data, this and similar surveillance data should be aggregated and proper attribution should be given to countries that share this data. Data for sub-national sites should be provided where possible and where appropriate to do so. Given its importance to global estimates, efforts should also be made to routinely publish the sub-national Indian NRSN data points in an accessible spreadsheet format, with data points presented by sub-national location and data year. Other partners and networks that generate rotavirus-positive proportions are also encouraged to share their data in an accessible format, wherever possible.

### Extrapolation of data points to country-level estimates using methods that appropriately capture between-country variation

Statistical regression modelling or finer levels of stratification than geographical region should be used when and where possible to extrapolate sub-national data points to different countries. The source of all sub-national, national and supranational indicators used to inform statistical regression models (e.g. GDP per capita) should be clearly defined. Care should be taken to ensure the coverage and impact of rotavirus vaccination is consistently captured at all levels of the analysis, including U5 deaths, the proportion of U5 deaths due to diarrhoea, and the proportion of U5 diarrhoea deaths due to rotavirus. Where possible, groups should extract and test the importance of other potentially influential sub-national characteristics e.g. private/public hospital, secondary/tertiary hospital, under-five mortality rate, proportion of patients from rural areas etc. If it is not feasible to collect this information from all sites, then a more detailed review of the GRSN dataset could be informative, and would allow comparison of several sub-national sites within the same countries. Input data points should be disaggregated into individual years of data collection to capture changes in the rotavirus-positive proportion over time at country level; covariates linked to period effects (e.g. GDP per capita, under-five mortality rate) should be carefully selected on the basis of their ability to reproduce observed trends in the rotavirus-positive proportion.

### Separation of the clinical syndromes of U5 diarrhoea hospitalisations

Rotavirus is not associated clinically with acute bloody or persistent diarrhoea, two diarrhoeal syndromes that may also be proximal or distal causes of death. Rotavirus-positive proportions derived exclusively from acute watery U5 diarrhoea hospitalisations should be adjusted to account for the proportion of total U5 diarrhoea hospitalisations that are acute watery. If the rotavirus-positive proportion (*r*) is not reported for all clinical syndromes combined, then the equation *r* = *ab* + *c*(1 − *b*) can be used, where *a* is the rotavirus-positive proportion among acute watery U5 diarrhoea hospitalisations, *b* is the proportion of total U5 diarrhoea hospitalisations that are acute watery, and *c* is the rotavirus-positive proportion among acute bloody and persistent U5 diarrhoea hospitalisations combined. In the absence of local data to inform parameter *b*, our analysis shows that acute watery diarrhoea is likely to be responsible for no more than 87% (95% CI 83–90%) of U5 diarrhoea hospitalisations. The true value of *b* is likely to be lower because all data points included in the meta-analysis under-estimated the role of persistent diarrhoea. Given that rotavirus is not associated clinically with acute bloody or persistent diarrhoea, the value of parameter *c* is likely to be at least ~3% based on the rotavirus-positivity observed in healthy controls in GEMS.

### Accounting for uncertainty in the steps used to convert rotavirus-positive proportions into rotavirus-attributable fractions

The frequent asymptomatic carriage of many pathogens in the stools of healthy controls necessitates the calculation of attributable fractions. To estimate the proportion of rotavirus-positive cases that are attributable only to rotavirus, the population attributable fraction estimated by the equation *r* × *AFe* can be used, where *r* is the rotavirus-positive proportion reported among U5 diarrhoea hospitalisations (all syndromes combined), and *AFe* is the rotavirus-attributable fraction among rotavirus-positive U5 diarrhoea hospitalisations (all syndromes combined). Because it is rare for diarrhoea surveillance studies to include diarrhoea-free controls, very few studies allow calculation of *AFe*. GEMS does include diarrhoea-free controls so permits this calculation; our new analysis of GEMS calculated the AFe to be 0.97 (95% CI 0.95–0.98). This value was relatively consistent across all GEMS sites where it could be reported (Bangladesh, India, Gambia, Kenya). This suggests that rotavirus is the attributable cause in almost all U5 rotavirus-positive diarrhoea hospitalisations. In a separate, related analysis, the rotavirus attributable fraction was shown to increase by a factor of 1.08 (95% 1.02–1.14) when the more sensitive qPCR test was used. This is similar (albeit slightly larger) than the adjustment made to *r* to account for *AFe*, so both adjustments could reasonably be excluded, and this would have a limited impact on central estimates of U5 rotavirus deaths. However, adjustments applied to some pathogens and not others, would lead to inconsistent reporting of central estimates (and uncertainty intervals) across enteric pathogens. These adjustments, and their uncertainty, should therefore be reflected in future estimates for all enteric pathogens, including rotavirus.

### Further research into the clinical syndromes of U5 diarrhoea deaths, and the real-world impact of rotavirus vaccines on those deaths

To date, all groups have assumed that the proportion of U5 diarrhoea hospitalisations caused by rotavirus is a reasonable proxy for the proportion of U5 diarrhoea deaths caused by rotavirus. This approach has been taken because hospitalisation is thought to be a good proxy for diarrhoea that is sufficiently severe to lead to death. Two aspects of our analysis suggest this assumption may lead to over-estimates of the number of U5 rotavirus deaths. First, we estimate that acute watery diarrhoea is associated with 87% of diarrhoea hospitalisations but only 65% of U5 diarrhoea deaths. Higher case fatality ratios (CFR) have been reported for acute bloody and persistent diarrhoea than acute watery diarrhoea [23] but more evidence on the fatality of different syndromes is needed to corroborate this. In addition, the analysis of diarrhoeal deaths relied on verbal autopsy reports which may be prone to recall bias, and our analysis of diarrhoea hospitalisations only included those that became persistent after admission. Second, rotavirus was detected in a higher proportion of U5 acute watery diarrhoea hospitalisations than U5 acute watery diarrhoea deaths in GEMS (44% vs 28%). Thus, among children that had access to treatment, rotavirus was estimated to be less fatal than other causes of acute watery diarrhoea. However, more evidence is needed on the effect of treatment on the proportion of acute watery diarrhoea deaths due to rotavirus; in communities without access to treatment services, rotavirus may represent a larger proportion of acute watery diarrhoea deaths. Another explanation for the lower rotavirus-positivity among U5 acute watery diarrhoea deaths, is that the number of deaths captured in the 7 days after enrolment (n = 37) were too few to make a reliable assessment. Longer follow-up periods allow more deaths to be included but it then becomes increasingly difficult to ascertain whether children who were rotavirus-positive at the time of enrolment were still rotavirus-positive at the time of death, and whether cases that were negative at enrolment had a new rotavirus episode prior to death.

Further evidence is needed from other geographical locations on the distribution of clinical syndromes among U5 diarrhoea hospitalisations and deaths. This should include a more accurate assessment of the role of persistent diarrhoea among U5 diarrhoea hospitalisations. More importantly, efforts should be made to accurately capture the real-world impact of rotavirus vaccines on U5 diarrhoea deaths in early introducing countries. This will provide critical insights into the true contribution of rotavirus to U5 diarrhoea deaths in different locations.

### Presenting and incorporating the uncertainty in parameters used to derive U5 rotavirus deaths

The uncertainty interval around the central estimates of U5 rotavirus deaths should be explicitly defined (e.g. the type of confidence or prediction interval) and should incorporate uncertainty in each of the three core parameters (number of U5 deaths, % due to diarrhoea, % due to rotavirus) as well as any other parameters used to adjust the original input data points e.g. the parameters used to convert rotavirus-positive proportions into rotavirus-attributable fractions.

## Conclusion

**Fig 5:**
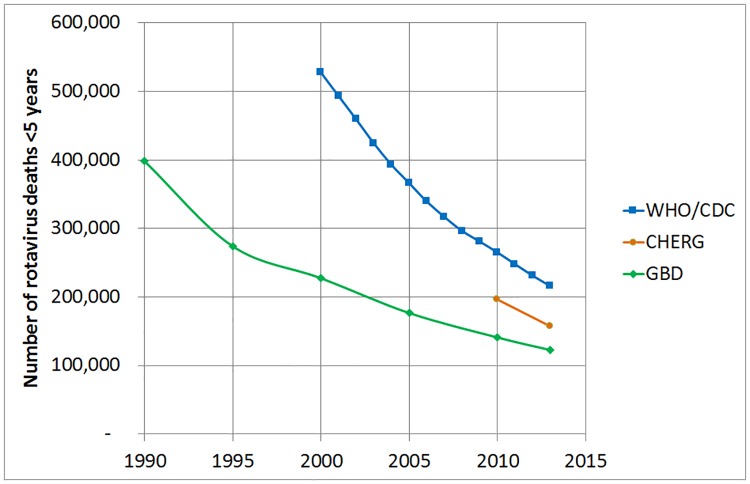
Global estimates of the number of rotavirus deaths <5 years in the year 2013 by source of estimates.

There is considerable disagreement between global estimates of U5 rotavirus deaths, but it is encouraging to note that estimates are converging over time, at least in absolute terms (Fig 5).

The aim of this analysis was not to recommend a single set of best estimates, but rather to explain the reasons for differences, provide evidence to inform key areas of uncertainty, and propose improvements for future estimates. Updates to GBD [24] and CHERG (now MCEE) estimates were already well advanced during the course of this comparison study, and further convergence is expected. The suggested improvements presented in this manuscript should be incorporated, as far as possible, into future rotavirus mortality estimates. This is likely to be an iterative and evolving process as new evidence emerges over time.

## Supporting information

S1 Table Information about the data used for new analyses. (DOCX)

S1 Appendix Further details on the comparison of rotavirus mortality estimates from GBD, CHERG and WHO/CDC. (DOCX)

S1 File Country-level dataset used to compare CHERG, GBD and WHO/CDC estimates of U5 deaths, U5 diarrhoea deaths and U5 rotavirus deaths in the year 2013. (XLSM)

## Abbreviations

AF: Attributable fraction
AFe: Attributable fraction in the exposed
AWD: Acute watery diarrhoea
CHERG: Child Health Epidemiology Reference Group of UNICEF and the World Health Organization (now MCEE—Maternal Child Epidemiology Estimation Group)
EIA: Enzyme Immunoassay
GBD: Global Burden of Disease Study
GEMS: Global Enteric Multicenter Study
GRSN: WHO-coordinated Global Sentinel Site Rotavirus Surveillance Network
IGME: UN Inter-agency Group for Child Mortality Estimation
MAL-ED: Malnutrition and Enteric Disease Study
MSD: Moderate-to-severe diarrhoea
NRSN: Indian National Hospital Rotavirus Surveillance Network
U5: Under-five years of age
WHO/CDC: World Health Organization and Centers for Disease Control and Prevention
