# Supplementary material for: Estimating global, regional and national rotavirus deaths in children aged <5 years: Current approaches, new analyses and proposed improvements
Source: PLoS One. 2017 Sep 11;12(9):e0183392. doi: 10.1371/journal.pone.0183392 (PMC5593200; doi:10.1371/journal.pone.0183392)
Supplement: S1 Table — (DOCX) [file pone.0183392.s001.docx]

**S1 Table. Information about the data used for new analyses**

| **New analysis** | **Where and how data was collected** | **Ethics statement** |
| --- | --- | --- |
| Clinical syndromes of U5 diarrhoea hospitalisations | WHO/CDC review of paediatric logbooks (Indonesia) and electronic discharge data (Rwanda and Zambia) from 50 hospitals included in the WHO-coordinated Global Sentinel Site Rotavirus Surveillance Network – GRSN. | All WHO GRSN surveillance data is from public health surveillance records. All data were anonymized prior to access and analysis. Institutional ethical approval was granted by CDC for the review of logbooks and analysis of the GRSN database. More details about GRSN are available at: <https://www.cdc.gov/mmwr/>  preview/mmwrhtml/mm6329a5.htm |
|  | CMC/CDC review of paediatric logbooks from 7 hospitals included in the Indian National Hospital Rotavirus Surveillance Network – NRSN. | All data were available under an existing disease burden project approved by the Institutional Review Board at the Christian Medical College (CMC) in Vellore. All data were anonymized prior to access and analysis. |
|  | All data were collected as part of the previously published Global Enteric Multicenter Study – GEMS (Kotloff K. Lancet 2013). Data were from 27 hospitals from 5 country sites included in GEMS. | GEMS was approved by the ethics committee at the University of Maryland, Baltimore, MD, USA, and at every field site. Written informed consent was obtained from the parent or primary caretaker of each participant before initiation of study activities. All data were anonymized prior to access and analysis. Datasets are available upon request from: <http://www.medschool.umaryland.edu/>  CVD/Projects/Global-Enteric-Multicenter-Study-GEMS/ |
| Clinical syndromes of U5 diarrhoea deaths | Investigation of a representative sample of child deaths identified in a household survey, including deaths in the community or a health facility, using the birth history method with follow-up questions to the mother or caregiver of the deceased child. Verbal autopsy data collected as part of national or multi-district verbal/social autopsy (VASA) studies in Cameroon (18,000 households in 2012), Malawi (24,000 households in 2012), Niger (25,000 households in 2010) and Nigeria (40,680 households in 2014). For more details please see Adewemimo A. et al (PLOS One 2017)  <http://journals.plos.org/plosone/>  article?id=10.1371/journal.pone.0178129. | All four VASA studies underwent ethical review and were approved by the Johns Hopkins University Institutional Review Board and the respective national ethical review committee of each study country. All data were anonymized prior to access and analysis. Nigeria data available from: <https://doi.org/10.5281/zenodo.569864>  Niger data available upon request from the National Institute of Statistics (INS – Niger):  [http://www.stat-niger.org/nada](http://www.stat-niger.org/nada /index.php/catalog/70#page =accesspolicy&tab=study-desc)  [/index.php/catalog/70#page](http://www.stat-niger.org/nada /index.php/catalog/70#page =accesspolicy&tab=study-desc)  [=accesspolicy&tab=study-desc](http://www.stat-niger.org/nada /index.php/catalog/70#page =accesspolicy&tab=study-desc) |
| Rotavirus-positive proportion in U5 diarrhoea hospitalisations and U5 diarrhoea deaths in GEMS | All data were collected as part of the previously published Global Enteric Multicenter Study – GEMS (Kotloff K. Lancet 2013). Data from 6 of the 7 country sites included in GEMS. | GEMS was approved by the ethics committee at the University of Maryland, Baltimore, MD, USA, and at every field site. Written informed consent was obtained from the parent or primary caretaker of each participant before initiation of study activities. All data were anonymized prior to access and analysis. Datasets are available upon request from:  <http://www.medschool.umaryland.edu/>  CVD/Projects/Global-Enteric-Multicenter-Study-GEMS/. The verbal autopsy data from GEMS are not publicly available. These data are collected by the site’s Demographic Surveillance System (DSS) and were shared with GEMS. Thus the sharing, but not the procedure, was described in the GEMS protocol. All data were anonymized prior to access and analysis. |
| Rotavirus attributable fraction among rotavirus-positive U5 diarrhoea hospitalisations in GEMS | All data were collected as part of the previously published Global Enteric Multicenter Study – GEMS (Kotloff K. Lancet 2013). Data were from 6 of the 7 country sites included in GEMS. | GEMS was approved by the ethics committee at the University of Maryland, Baltimore, MD, USA, and at every field site. Written informed consent was obtained from the parent or primary caretaker of each participant before initiation of study activities. All data were anonymized prior to access and analysis. Datasets are available upon request from:  <http://www.medschool.umaryland.edu/>  CVD/Projects/Global-Enteric-Multicenter-Study-GEMS/ |
