# Supplementary material for: Estimating global, regional and national rotavirus deaths in children aged <5 years: Current approaches, new analyses and proposed improvements
Source: PLoS One. 2017 Sep 11;12(9):e0183392. doi: 10.1371/journal.pone.0183392 (PMC5593200; doi:10.1371/journal.pone.0183392)
Supplement: S1 Appendix — (DOCX) [file pone.0183392.s002.docx]

**S1 Appendix. Further details on the comparison of rotavirus mortality estimates from GBD, CHERG and WHO/CDC**

GBD, CHERG and WHO/CDC used different methods to:

1. select data points (rotavirus-positive proportions);
2. extrapolate data points to individual countries;
3. account for rotavirus vaccine coverage;
4. convert rotavirus-positive proportions to rotavirus attributable fractions; and,
5. calculate uncertainty ranges.

The following provides a fuller description of these differences:

**Data points (rotavirus-positive proportions)**

CHERG identified 242 data points (rotavirus-positive proportions) from 76 countries; GBD identified 1336 data points from 71 countries; and, WHO/CDC identified 774 data points from 90 countries. 110 countries were included by at least one of the three sources, and only 42 were identified by all three. There was considerable variation in the combination of other countries included by each source (see Table overleaf).

**S1 Appendix Table. Number of countries with data points (rotavirus-positive proportions <5 years) included by CHERG, GBD and WHO/CDC by WHO region**

| **WHO region** | **CHERG** | **GBD** | **WHO /CDC** | **All three sources** | **At least one source** |
| --- | --- | --- | --- | --- | --- |
| AFRO | 15 | 14 | 20 | 8 | 25 |
| AMRO | 10 | 11 | 18 | 8 | 19 |
| EMRO | 11 | 8 | 16 | 5 | 16 |
| EURO | 23 | 23 | 18 | 9 | 31 |
| SEARO | 6 | 5 | 7 | 4 | 7 |
| WPRO | 11 | 10 | 11 | 8 | 12 |
| **GLOBAL** | **76** | **71** | **90** | **42** | **110** |

Where possible, GBD and WHO/CDC disaggregated studies into sub-national sites and 12-month periods, and entered each as a separate data point.

All three groups excluded any data points that did not represent at least 12 months of data, thus avoiding seasonality issues. However, the groups differed on other inclusion and exclusion criteria. CHERG and WHO/CDC did not include rotavirus proportions derived from outpatients, but GBD included data points for both outpatients and inpatients, and made an explicit adjustment for inpatient status in their regression model. 33 of the 71 countries included by GBD had rotavirus-positive proportions for both inpatients and outpatients, 6 countries had data points for outpatients only (Austria, Denmark, Germany, Iceland, Saudi Arabia, Zambia) and 32 countries had data points for inpatients only. CHERG and GBD included data points from studies published from January 1990 onwards. WHO/CDC only included data points with a mid-year data collection point from July 1998 onwards. GBD and WHO/CDC excluded data points based on fewer than 100 tested diarrhoea samples but CHERG did not set a lower threshold for this. WHO/CDC only included studies that reported data points for the entire <5 year age range whereas both CHERG and GBD included data points reported for narrower age bands (e.g. <2yrs) and made adjustments to account for missing data. GBD did not use GRSN data, but this was the only source of evidence used in 20 of the 32 GRSN countries included by CHERG and 16 of the 61 GRSN countries included by WHO/CDC.

CHERG found 39% rotavirus positivity in 180 single-pathogen inpatient studies compared to 20% rotavirus-positivity in 24 inpatient studies that tested for at least 5 pathogens [1]. This suggests a bias associated with single-pathogen rotavirus studies. Rotavirus studies may be more likely to be conducted in areas with higher rotavirus prevalence, and more likely to exclude acute bloody and persistent diarrhoea cases, leading to inflated estimates of the rotavirus-positive proportion. To allow further investigation of this bias, future estimates should report the number of pathogens tested for each data point used.

The methods used by WHO/CDC and CHERG are relatively straightforward and derived from data points that are in general, publicly available. WHO routinely publishes summary reports of the GRSN data, but it is not currently possible to download this information in an editable spreadsheet format. Greater access to this information would permit its use by all groups in future estimates. The methods used by GBD are more complex and include a small proportion of data points that are not available publicly. In an effort to increase transparency, GBD will release all of its computer codes in future GBD releases. They do however acknowledge that it would be very difficult for others to replicate their estimates without intimate understanding of their source files and code [2].

Recent Guidelines for Accurate and Transparent Health Estimates Reporting (GATHER) have recommended publication of a spreadsheet table with details about the data points used to inform estimates [3]. We recommend a set of minimum variables that should be included in future estimates (see first recommendation in main manuscript). The GATHER checklist also requires detailed documentation of inclusion and exclusion criteria and methods of data analysis, and should help to increase transparency in reporting of methods in future updates.

**Extrapolating rotavirus-positive proportions to individual countries**

Different methods are used to extrapolate sub-national data points to individual countries. WHO/CDC and GBD use regression models. CHERG calculates a median for each region and then extrapolates this to all countries within each region. Covariates used by WHO/CDC were the calendar year, Millennium Development Goal (MDG) region, national under-five mortality rate and national rotavirus vaccine coverage level. GBD use their *Dismod-MR* regression model, a Bayesian, hierarchical, mixed-effects meta-regression model with fixed effects for sex, inpatient sample status and broad screening method and random effects to account for super-region, region, country, age and calendar year.

All three groups assumed that sub-national data points were representative of the national situation. However, most GRSN sites and most other sites reported in the published literature are located in major urban areas where socioeconomic conditions, environmental risks and access to treatment may be different to the rest of the country. Issues of representativeness can be overcome if regression models include characteristics that are specific to each sub-national data point e.g. proportion of patients from rural areas, under-five mortality rate, private/public hospital, secondary/tertiary hospital etc. However, both GBD and WHO/CDC used national variables to define sub-national data points when constructing their respective regression models. This is a reasonable approach in the absence of detailed sub-national information about each site. However, where possible, groups should extract and test the importance of other potentially influential sub-national characteristics e.g. private/public hospital, secondary/tertiary hospital, under-five mortality rate, proportion of patients from rural areas etc. If it is not feasible to collect this information from all sites, then a more detailed review of the GRSN dataset could be informative, and would allow comparison of several sub-national sites within the same countries.

WHO/CDC restricted their dataset to data points collected after July 1998 because previous literature reviews had shown an increase in the rotavirus-positive proportion from 22% (for studies published 1986-1999) to 39% (for studies published 2000-2004). This warrants further scrutiny. In particular, whether the trend is still observed after correcting for the improved sensitivity of testing in more recent years (e.g. rectal swabs vs EIA) and the increased number of single-pathogen rotavirus studies conducted in more recent years. These single-pathogen studies were shown by CHERG to give a significantly higher rotavirus-positive proportion than multiple pathogen studies. If such a trend were to exist after correcting for these potential biases, then regression models could explicitly account for the period of data collection. Period effects may be closely linked to the stage of economic development in each country, and thus may already be captured by existing covariates e.g. GDP per capita, under-five mortality rate etc. However, estimates for historical years published by GBD (1990-2013) and WHO/CDC (2000-2013) indicate very little change in the proportion of U5 diarrhoea deaths caused by rotavirus each year. This suggests the effect is either not observed in the datasets, or does exist, and is not appropriately captured in the regression models.

**Accounting for rotavirus vaccine coverage**

CHERG did not account for vaccine coverage in their 2010 estimates because very few countries had introduced rotavirus vaccines before that year. For the purpose of this comparison exercise, the pre-vaccine rotavirus-positive proportions reported by CHERG for 2010 were applied to pre-vaccine era U5 diarrhoea deaths estimated by CHERG for 2013, and no adjustment was made for the small number of countries that had introduced rotavirus vaccination before 2013. To estimate U5 diarrhoea deaths in the year 2013, GBD included rotavirus vaccine introduction status as a binary covariate in their *CODEm* regression model. However, the rotavirus-attributable fractions used by GBD for years with rotavirus vaccination, were based on data points (rotavirus-positive proportions) extracted from the pre-vaccine era. Vaccine-adjusted estimates of U5 diarrhoea deaths were therefore combined with rotavirus-attributable fractions that had not been adjusted for rotavirus vaccine use. WHO/CDC included a binary covariate in their regression model used to predict the rotavirus-positive proportion. This covariate was used to differentiate sites that had introduced rotavirus vaccines to a reasonable level of coverage (>60% coverage of children aged <1 year, at least 12 months after vaccine introduction). However, the 2000-2013 CHERG estimates of U5 diarrhoea deaths available at the time of the analysis were not adjusted for rotavirus vaccine use [4]. WHO/CDC therefore combined U5 diarrhoea deaths that had not been adjusted for vaccine use, with rotavirus-positive proportions that had been. In summary, both GBD and WHO/CDC estimates were adjusted for rotavirus vaccine impact at one level of the analysis but not both, and this may have led to over-estimation of rotavirus deaths in a small number of countries that had introduced rotavirus vaccination in 2013. Including a rotavirus vaccine coverage covariate at both levels of the analysis (U5 diarrhoea deaths, rotavirus-positive proportion) would partly overcome this problem. However, if separate regression models or separate sources of estimates are used at each level of the analysis, this may lead to estimates of U5 diarrhoea deaths and U5 rotavirus deaths that are not internally consistent in post-vaccination years. This could be overcome by generating a single estimate of the number of rotavirus deaths prevented by vaccination, and adjusting both the number of U5 diarrhoea deaths and the number of U5 rotavirus deaths by the same consistent number. Joint WHO/UNICEF estimates of coverage have been standardised across countries (accounting for both household surveys and administrative data) and should ideally be used for these adjustments.

**Converting rotavirus-positive proportions to rotavirus attributable fractions**

WHO/CDC used the rotavirus-positive proportion as a direct proxy for the rotavirus attributable fraction, and did not make further adjustments.

CHERG added together: (a) the median rotavirus-positive proportion, based on all included data points; (b) the median pathogen-positive proportions reported for all other enteric pathogens, based on studies that sought 5-13 pathogens (n=27); and, (c) the median proportion of stool samples with unknown etiology, based on 12 studies that sought at least 8 pathogens. The total summed to greater than 100%, so all medians were rescaled to 100%. Pathogen-positive proportions were therefore converted into something closer to pathogen-attributable fractions. The rescaling of pathogen-positive proportions is one approach to attributing mixed infections to a single cause, but this does not account for differences in pathogenicity. Re-analysis of diarrhoeal samples from GEMS using a pan-molecular approach with real-time, quantitative PCR [5, 6] suggests that the pathogenicity of different organisms is very different. The low prevalence of rotavirus identified in the stools of healthy controls compared to diarrhoea hospitalisations in GEMS (3% vs 38% - See main manuscript, Table 3) suggests that rotavirus is likely to be an important cause of severe diarrhoea whenever it is detected. A similar pattern (6% vs 27%) was also observed in the Malnutrition and Enteric Disease Study (MAL-ED) where children from 8 sites in South America, Africa and Asia were followed from birth until age 24 months [7]. The proportion of samples with unknown etiology (34%) was also included in the CHERG rescaling process, but this did not account for variability in the performance of the conventional tests used to identify each pathogen. Retesting of stool samples from the GEMS case control study using the Polymerase Chain Reaction (PCR) TaqMan® array card recently found significant variation in test performance [8]. It should be noted that the results of the new PCR analyses were not available at the time the 2010 CHERG estimates were developed.

GBD derived rotavirus-attributable fractions by multiplying rotavirus-positive proportions by 1/(1-OR), where OR is the odds ratio derived from the GEMS case control study, and reflects the odds of having MSD if rotavirus is detected in the stool using the conventional EIA test. ORs were calculated for all enteric pathogens included in GEMS. The average ORs of countries with GEMS sites in African and Asian sub-regions were applied to other countries in that sub-region. The average ORs across all GEMS sites was applied to all other countries. The original GEMS analysis [9] first calculated odds ratios, and then adjusted for the presence of other pathogens using the Bruzzi correction [10]. GBD derived their own ORs from the GEMS dataset; these ORs did not account for socio-demographic characteristics but did account for inter-site variability [11]. In both the primary GEMS analysis and GBD reanalysis the ORs were calculated using MSD cases, which include both outpatients and inpatients. GBD apply attributable fractions for each pathogen to diarrhoea deaths and any remaining deaths not assigned to a cause are considered to be unknown. The number of diarrhoea deaths with an unknown cause was around 40% in GBD 2013 [11].

**Uncertainty**

WHO/CDC estimates include uncertainty in the proportion of U5 diarrhoea deaths due to rotavirus, but not higher level uncertainty in U5 deaths and U5 diarrhoea deaths. Both CHERG and GBD generate probabilistic uncertainty intervals that account for uncertainty in U5 deaths, the proportion due to diarrhoea, and the proportion due to rotavirus. In addition, GBD includes uncertainty in the GEMS odds ratios applied to rotavirus-positive proportions. CHERG separately presented uncertainty that was due to parameter inputs versus specific methodological choices e.g. they showed deaths with and without the inclusion of single-pathogen studies.

**References for S1 Appendix**

1. Lanata CF, Fischer-Walker CL, Olascoaga AC, Torres CX, Aryee MJ, Black RE. Global causes of diarrheal disease mortality in children <5 years of age: a systematic review. PLoS One. 2013;8(9):e72788.

2. Theo Vos RB, David E Phillips, Alan D Lopez, Christopher J L Murray. Authors Reply to Correspondence - Causes of child death: comparison of MCEE and GBD 2013 estimates. Li Liu, Robert E Black, Simon Cousens, Colin Mathers, Joy E Lawn, Daniel R Hogan. Published Online May 23, 2015 <http://dx.doi.org/10.1016/> S0140-6736(15)60663-8.

3. Stevens GA, Alkema L, Black RE, Boerma JT, Collins GS, Ezzati M, et al. Guidelines for Accurate and Transparent Health Estimates Reporting: the GATHER statement. Lancet. 2016.

4. World Health Organization. Child cause of death estimates 2000-2013. Available at: http://www.who.int/entity/healthinfo/global_burden_disease/childCOD_estimates_2000_2013.xls?ua=1. Accessed 11 January 2017.

5. Liu J, Kabir F, Manneh J, Lertsethtakarn P, Begum S, Gratz J, et al. Development and assessment of molecular diagnostic tests for 15 enteropathogens causing childhood diarrhoea: a multicentre study. Lancet Infect Dis. 2014;14(8):716-24.

6. Platts-Mills JA, Gratz J, Mduma E, Svensen E, Amour C, Liu J, et al. Association between stool enteropathogen quantity and disease in Tanzanian children using TaqMan array cards: a nested case-control study. Am J Trop Med Hyg. 2014;90(1):133-8.

7. Platts-Mills JA, Babji S, Bodhidatta L, Gratz J, Haque R, Havt A, et al. Pathogen-specific burdens of community diarrhoea in developing countries: a multisite birth cohort study (MAL-ED). Lancet Glob Health. 2015;3(9):e564-75.

8. Liu J, Platts-Mills JA, Juma J, Kabir F, Nkeze J, Okoi C, et al. Use of quantitative molecular diagnostic methods to identify causes of diarrhoea in children: a reanalysis of the GEMS case-control study. Lancet. 2016;388(10051):1291-301.

9. Blackwelder WC, Biswas K, Wu Y, Kotloff KL, Farag TH, Nasrin D, et al. Statistical methods in the Global Enteric Multicenter Study (GEMS). Clin Infect Dis. 2012;55 Suppl 4:S246-53.

10. Bruzzi P, Green SB, Byar DP, Brinton LA, Schairer C. Estimating the population attributable risk for multiple risk factors using case-control data. Am J Epidemiol. 1985;122(5):904-14.

11. Global, regional, and national age-sex specific all-cause and cause-specific mortality for 240 causes of death, 1990-2013: a systematic analysis for the Global Burden of Disease Study 2013. Lancet. 2015;385(9963):117-71.
